# Deciding the operation type according to mismatch repair status among hereditary nonpolyposis colorectal cancer patients: should a tailored approach be applied, or does one size fit all?

**DOI:** 10.1186/s13053-021-00186-x

**Published:** 2021-06-29

**Authors:** Chun-Kai Liao, Yueh-Chen Lin, Yu-Jen Hsu, Yih-Jong Chern, Jeng-Fu You, Jy-Ming Chiang

**Affiliations:** 1 Colorectal Section, Department of Surgery, Chang Gung Memorial Hospital, No. 5, Fuxing St., Guishan Dist,, Taoyuan, Taiwan 33305; 2 College of Medicine, Chang Gung University, Taoyuan, Taiwan

**Keywords:** HNPCC, MMR status, Extended colectomy, Metachronous CRC, Overall survival

## Abstract

**Background:**

Although extended colectomy (EC) was recommended for HNPCC patients, previous studies did not show significantly improved overall survival. Immunohistochemical (IHC) stain of mismatch repair (MMR) gene protein expression is now a feasible and reliable test clinically. Therefore, we tried to investigate whether we could use MMR IHC stain to select operation types in HNPCC patients.

**Patients and methods:**

Between 1995 and 2013, 186 HNPCC patients were collected. Status of MMR protein expression, perioperative clinic-pathological variables and post-operative follow up status were analyzed by multivariate analyses.

**Results:**

Sixty-five percent (121 of 186) patients of these HNPCC patients demonstrated loss of at least one MMR protein. There were several significant differences existing between deficient MMR (dMMR) and proficient MMR (pMMR) subgroups in terms of clinic-pathological characteristics. With the average follow-up duration of 93.9 months, we observed significantly high risk of developing metachronous CRC between SC and EC subgroups (crude rate 8.5% vs. 0%, *p* = 0.035). However, no significant difference was observed among the presence of extra-colonic tumors (12.4% vs. 5.8%, *p* = 0.284). The positive and negative prediction rate of metachronous CRC in dMMR subgroup was 12.8 and 87.2% while 1.9 and 98.1% in the pMMR subgroup.

Survival outcomes were significantly affected by MMR status and resection types by multivariate analysis. Significantly better OS in dMMR subgroup (HR = 0.479, 95% CI: 0.257–0.894, *p* = 0.021) comparing with pMMR subgroup was observed. However, significant improved DFS (HR = 0.367, 95% CI: 0.172–.0787, *p* = 0.010) but not significant for OS (HR = 0.510, 95% CI: 0.219–1.150, *p* = 0.103) for EC subgroup compared with SC subgroup.

Differences existing among different subgroups by combing extent of resection and MMR status. In dMMR subgroup, SC, compared with EC, demonstrated significantly worse DFS by multivariate analyses (HR = 3.526, 95% CI: 1.346–9.236, *p* = 0.010) but not for OS (HR = 2.387, 95% CI: 0.788–7.229, *p* = 0.124), however, no significantly differences of OS and DFS in pMMR subgroup between SC and EC were found.

**Conclusions:**

Significantly better overall survival and higher rate of metachronous CRC exist in dMMR subgroup of HNPCC patients comparing with pMMR subgroup. Extended colectomy significantly improved DFS and was thus recommended for dMMR subgroup but not pMMR subgroup of HNPCC patients.

## Background

Hereditary nonpolyposis colorectal cancer (HNPCC) patients defined by Amsterdam criteria clinically. These patients were characterized with increased risks of metachronous colorectal tumors [1] and extra-colonic cancers [2, 3], in addition to younger age at onset, right side colon predominance, higher proportion of poorly differentiated and mucinous adenocarcinoma [2, 4, 5].

To improve surgical outcomes of HNPCC patients, extended colectomy (EC), such as subtotal or total colectomy, rather than segmental colectomy (SC) or hemicolectomy, is recommended owing to the nature of increased risks of metachronous colorectal tumors among HNPCC patients [6–11]. Following these recommended guidelines has showed to decrease the rate of metachronous colorectal lesions significantly [1, 6–11], however, the survival rate seems not significantly improved for extended colectomy subgroups [7, 11, 12]. The reasons may be resulted from adequate post colectomy colonoscopy surveillance or these cancers are detected at an early stage. In contrast, functional results and quality of life post extended colectomy (EC) was concerned with frequency, urgency and varied degrees of stool incontinence [13]. Therefore, the optimal operation type among HNPCC patients remains controversial.

Universal testing of all newly diagnosed CRC patients was considered to be practicable currently and tested for deficient mismatch repair (dMMR) protein expression status may be useful in clinical practice [14] such as to determine post-operative adjuvant chemotherapy for stage III patients [15] or identification of **Lynch** syndrome patients from familial colorectal cancer type X (FCCTX) patients that have distinct clinical behavior **form** [16–18]. However, the value of pre-colectomy status of MMR expression related to surgical treatment of HNPCC patients remained unclear. In this retrospective study, we analyzed 186 HNPCC patients to investigate whether MMR expression status may help decide types of colectomy among these patients and to achieve improved overall and disease-free survival.

## Material and methods

### Patient selection

Between January 1995 and September 2013, a total of 186 consecutive patients fulfilled the Amsterdam criteria II (AC II) underwent colectomy for colorectal cancer at Chang Gung Memorial Hospital (CGMH) were retrospectively analyzed. Patients fulfilling the AC II (at least three relatives with a Lynch-associated cancer, one being a first-degree relative of the other two; at least two successive generations affected; and at least one person diagnosed before 50 years of age) were defined as HNPCC patients. The study was approved by the IRB of Chang Gung Memorial Hospital (IRB201801201B).

### Data collection

A retrospective analysis of data from the Colorectal Section Tumor Registry in Chang Gung Memorial Hospital, a prospectively designed database consisting of the records of postoperative patients who were consecutively and actively followed up, was conducted. Written informed consent was obtained from the patients prior to study participation. This study was approved by the Institutional Review Board of Chang Gung Memorial Hospital (approval number IRB102-2284B), and performed in accordance with the Declaration of Helsinki. All data were recorded in the hospital database and used for research purposes.

The peri-operative variables including age, sex, tumor location and surgery type were analyzed. The pathological parameters including MMR status, T-stage, N-stage, M-stage, histology grade and histology type were also analyzed.

### IHC analysis for MMR gene expression

Paraffin-embedded tumor blocks from HNPCC patients were retrieved from the Pathology Department of CGMH. For each patient, 4-μm thick sections from one formalin-fixed, paraffin-embedded tissue block containing both tumor tissue and normal adjacent mucosa were obtained. Immunostaining was performed on a Dako Universal Autostainer (DakoCytomation, Denmark) by using ChemMateTM EnvisonTM + Detection kits (DakoCytomation, Denmark) and described before [19]. For the evaluation of IHC results, abnormal staining was defined as total loss of protein in the tumor, using appropriate controls; staining was considered assessable when the nucleus was stained in cells serving as internal controls, including either stromal or germinal follicle lymphocytes or normal epithelial cells in the crypt bases. Tumors were considered negative for MMR protein expression when neoplastic cells showed complete absence of detectable nuclear staining in a sample for which internal positive controls were stained. Pathologists who had no knowledge of the family history or other clinicopathological features, reviewed all cases to confirm the immunostaining results.

### Follow up

All physicians at the author’s institution adopted similar follow-up routines and adjuvant treatment protocols. After primary tumor resection, all patients were subjected to a follow-up program that included outpatient visits every 3 to 6 months for physical examinations and carcinoembryonic antigen (CEA) tests. Chest radiography, abdominal ultrasonography, or abdominal computed tomography (CT) imaging, in addition to colonoscopy, were performed one year after the index surgery and then every 1 to 3 years whenever necessary. Follow-up status was confirmed postoperatively every 12 months by a team of three physicians and five specially trained nurses. Telephone interview or mail questionnaire was made if patient’s medical records were not available. Date of first recurrence was defined as the first date when the existence of local recurrence and/or distant metastases was confirmed by histology of biopsy specimens, additional surgery, and/or by radiological studies. The last follow-up date in this study was May 31, 2020.

### Statistical analyses

All parameters were analyzed using the Statistical Package for Social Sciences (SPSS) version 26 (IBM Corp., Armonk, New York). The categorical variables were compared using Pearson’s chi-squared test or Fisher’s exact test, whereas continuous variables were compared using the independent sample T-test. Survival analysis was performed using Kaplan-Meier curves with the log-rank test. The Cox proportional hazards model was used to investigate the effect of clinical variables on survival while adjusting for other explanatory variables. The *p*-values were two-sided and those < 0.05 were considered statistically significant.

## Results

In this cohort, a total of 186 patients fulfilled with Amsterdam criteria II were included. **As shown in** Table 1**, Sixty-five percent (121 of 186) patients of these HNPCC patients demonstrated loss of at least one MMR protein.** The distribution of the loss of MMR gene expression (shown in Table 2) included concordant losses of MLH1/PMS2 staining (82 of 121 patients, 67.8%), MSH2/MSH6 staining (35 of 121 patients, 28.9%) and loss of PMS2 only (4 of 121 patients, 3.3%). As shown in Table 1, there were several significant differences existing between dMMR and pMMR subgroups in terms of clinic-pathological characteristics. The mean age of diagnosis is younger in dMMR subgroup (47.5 vs. 54.6 years-old, *p* < 0.001). There is more extended colectomy in dMMR subgroup (Extended resection: 33.9% vs. 16.9%, *p* = 0.021). dMMR subgroup also presented with right colon predominance (right-sided colon: 61.2% vs. 33.8%, p < 0.001) and more N0 stage (N0 stage: 68.6% vs. 50.0%, *p* = 0.013).

**Table 1:** Basic characteristics and clinicopathological features among 186 HNPCC patients

	dMMR (*n* = 121)	pMMR (*n* = 65)	*p* Value
Age (mean ± SD), years	47.54 ± 12.39	54.62 ± 13.67	< 0.001
Sex			
Male	59 (48.8)	34 (52.3)	0.645
Female	62 (51.2)	31 (47.7)	
Cancer location			
Right colon	74 (61.2)	22 (33.8)	< 0.001
Left colon	30 (24.8)	20 (30.8)	
Rectum	17 (14.0)	23 (35.4)	
Histological type			
Adenocarcinoma	94 (77.7)	87 (87.7)	0.058*
Signet ring cell	2 (1.7)	2 (3.1)	
Mucinous	22 (18.2)	5 (7.7)	
NET	0 (0)	1 (1.5)	
Other	3 (2.5)	0 (0)	
Histological grade			
Grade I	17 (14)	10 (15.4)	0.048*
Grade II	70 (57.9)	47 (72.3)	
Grade III/IV	31 (25.6)	8 (12.3)	
Unclassified	3 (2.5)	0 (0)	
Operation type			
Segmental resection	78 (64.5)	51 (78.5)	0.021*
Extended resection	41 (33.9)	11 (16.9)	
Local resection	2 (1.7)	3 (4.6)	
T stage			
T0	2 (1.7)	1 (1.6)	0.397
T1	6 (5.0)	5 (7.8)	
T2	10 (8.3)	4 (6.3)	
T3	52 (43.0)	35 (54.7)	
T4	51 (42.1)	19 (29.7)	
N stage			
N0	83 (68.6)	32 (50)	0.013
N1	29 (24.0)	19 (29.7)	
N2	9 (7.4)	13 (20.3)	
M stage			
M0	10 (8.3)	6 (9.2)	0.823
M1	111 (91.7)	59 (90.8)	

*Fishers exact test

**Table 2:** Patterns of gene loss among 186 HNPCC patients

IHC staining pattern	n	Percentage
Absence of MLH1/PMS2	82	44.10%
Absence of MSH2/MSH6	35	18.80%
Absence of PMS2	4	2.20%
No loss	65	34.90%

The follow up status was summarized in Table 3. The average follow-up duration for total patients were 93.9 months, with 111.1 and 88.5 months in EC subgroup and SC subgroups respectively. In this cohort, we observed significantly high risk of developing metachronous CRC between SC and EC subgroups (crude rate 8.5% vs. 0%, *p* = 0.035). Time to developing metachronous CRC was average 39.6 months (range from 4.7 to 99.3 months). However, no significant difference was observed among the presence of extra-colonic tumors (12.4% vs. 5.8%, *p* = 0.284).

**Table 3:** Survival data of 181 HNPCC patients who underwent surgical resection of CRC

	Segmental resection (*n* = 129)	Extended resection (*n* = 52)	p Value
Follow-up time, months			
Mean	88.50 ± 43.92	111.10 ± 25.29	0.001
Median	119.54 (0.95–143.64)	121.05 (31.34–133.78)	
Survival, n (%)			
Dead	45 (34.9)	7 (13.5)	0.004
Alive	84 (65.1)	45 (86.5)	
Recurrence			
Presence	24 (18.6)	4 (7.7)	0.073
Absence	105 (81.4)	48 (92.3)	
Recurrence interval, months			
Mean	18.66 ± 14.12	49.29 ± 21.76	0.001
Median	11.86 (3.68–67.58)	54.8 (20.01–67.58)	
Metachronous colon lesions			
Presence	11 (8.5)	0 (0)	0.035*
Absence	118 (91.5)	52 (100)	
Recurrence interval, months			
Mean	39.64 ± 30.45		
Median	34.07 (4.70–99.35)		
Extracolonic lesion			
Presence	16 (12.4)	3 (5.8)	0.284
Absence	113 (87.6)	49 (94.2)	
Recurrence interval, months			
Mean	57.70 ± 41.60	76.16 ± 47.20	0.497
Median	51.78 (46.13–130.56)	30.62 (16.92–130.56)	

**Fig. 1:**
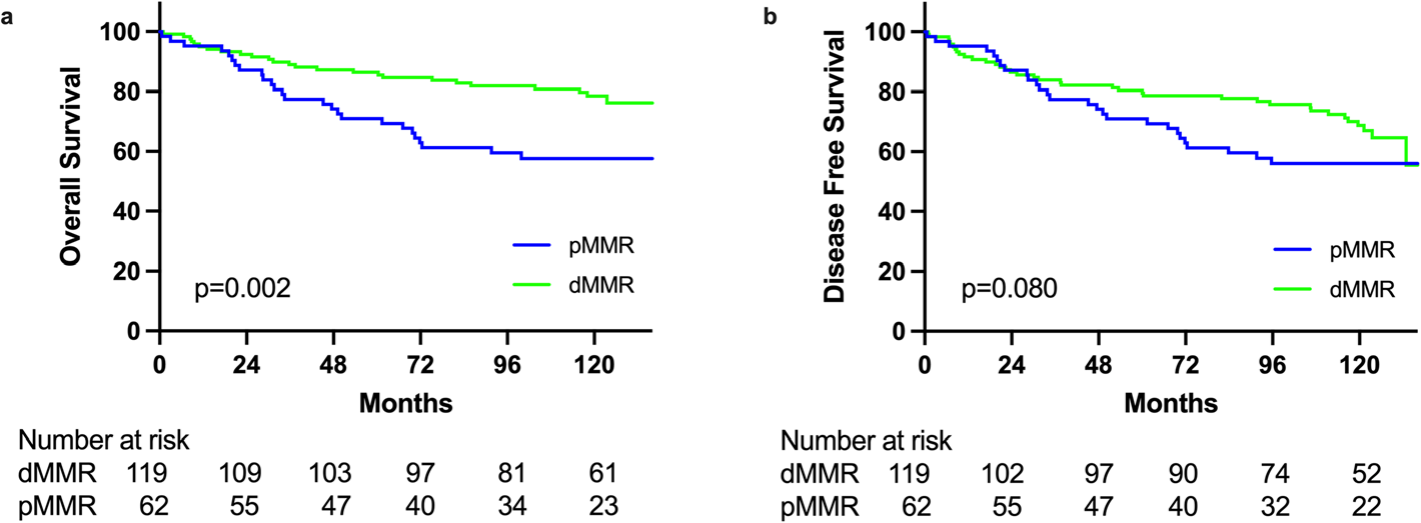
Comparison of overall survival (**a**) and disease-free survival (**b**) between patients with proficient mismatch repair gene (pMMR) and those with deficient mismatch repair gene (dMMR)

**Fig. 2:**
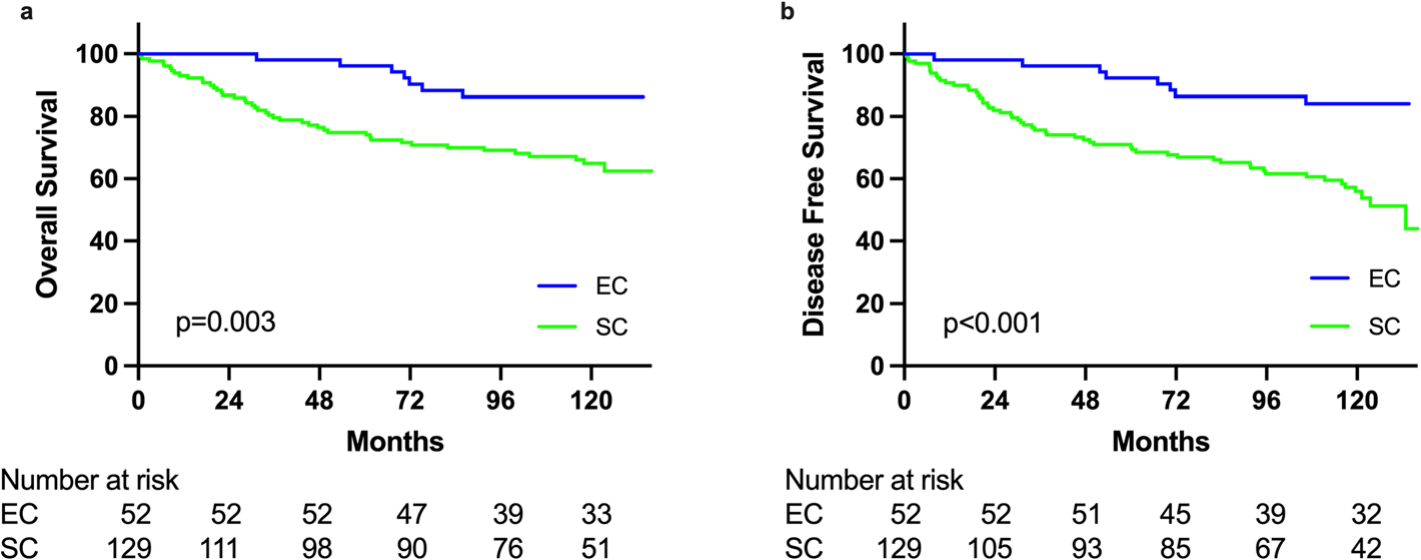
Comparison of overall survival (**a**) and disease-free survival (**b**) between patients who had extended colectomy (EC) and those who had segmental colectomy (SC)

Clinico-pathological factors related to OS (Table 4) and DFS (Table 5) were analyzed by univariate and multivariate analysis after excluding 5 patients without curative resection. **There were some significant factors existed relating to OS and DFS.** As shown in Table 4 and Table 5, in univariate analysis, MMR status showing a significantly better OS (*p* = 0.002) (Fig. 1A) but not DFS (*p* = 0.08) (Fig. 1B) for dMMR subgroup comparing with pMMR subgroup was observed. Multivariate analysis further confirmed significantly better OS in dMMR subgroup (HR = 0.479, 95% CI: 0.257–0.894, *p* = 0.021). Resection types also impact on surgical outcomes. Univariate analysis showed significantly better OS (*p* = 0.003) (Fig. 2A) and DFS (*p* < 0.001) (Fig. 2B) for EC subgroup comparing with SC subgroup. Multivariate analysis further confirmed significant DFS (HR = 0.367, 95% CI: 0.172–.0787, *p* = 0.010) but not significant for OS (HR = 0.510, 95% CI: 0.219–1.150, *p* = 0.103) for EC subgroup compared with SC subgroup.

**Table 4:** Univariate and multivariate analyses of clinicopathological features with OS among 181 HNPCC patients who underwent curative resection of CRC

Characteristics	n	OS (%)			p Value	Multiple Cox		
		1 year	3 years	5 years		HR	95% CI	p Value
Age, years								
< 40	42	95.2	90.3	90.3	0.05	1		
≥ 40	139	95	83.4	78.3		2.176	0.933–5.075	0.072
Sex								
Male	90	94.5	85.6	81.2	0.688			
Female	91	95.5	84.3	80.9				
MMR status								
pMMR	62	95.2	77.4	71	0.002	1		
dMMR	119	95	89	86.4		0.479	0.257–0.894	0.021
Tumor location								
Right colon	94	95.7	88.1	86	0.001	1		
Left colon	50	98	92	90		1.254	0.620–2.537	0.53
Rectum	37	89.2	67.6	56.8		1.302	0.628–2.702	0.478
Operation type								
Segmental resection	129	93	79.6	74.9	0.003	1		
Extended resection	52	100	98.1	96.2		0.501	0.219–1.150	0.103
Histological type								
Adenocarcinoma	146	95.9	84.8	79.9	0.993			
Signet ring cell/mucinous	31	93.5	87.1	87.1				
Other	4	75	75	75				
Histological grade								
Grade I	25	100	100	92	0.509			
Grade II	116	94	82.6	78.3				
Grade III/IV	38	94.7	81.2	81.2				
Unclassified	2	100	100	100				
T stage								
T0	3	100	100	100	0.073			
T1	10	100	100	80				
T2	14	92.9	92.9	92.9				
T3	86	98.8	84.5	82.1				
T4	68	89.7	80.9	76.5				
N stage								
N0	112	97.3	93.8	90.2	< 0.001	1		
N1	48	93.7	78.5	74.1		1.343	0.655–2.756	0.421
N2	21	85.7	52.4	47.6		3.605	1.623–8.009	0.002
M stage								
M0	167	97.6	89.1	86.1	< 0.001	1		
M1	14	64.3	35.7	19		7.723	3.362–17.742	< 0.001

**Table 5:** Univariate and multivariate analyses of clinicopathological features with DFS among 181 HNPCC patients who underwent curative resection for colorectal cancer

Characteristics	n	DFS (%)			p Value	Multiple Cox		
		1 year	3 years	5 years		HR	95% CI	p Value
Age, years								
< 40	42	95.2	90.3	87.9	0.081			
≥ 40	139	92.1	79	73.9				
Sex								
Male	90	92.2	79.8	76.4	0.967			
Female	91	93.4	83.4	77.8				
MMR status								
pMMR	62	95.2	77.4	71	0.08			
dMMR	119	91.6	83.9	80.5				
Tumor location								
Right colon	94	94.7	84.9	82.7	0.02	1		
Left colon	50	94	88	84		1.077	0.582–1.992	0.813
Rectum	37	86.5	64.9	54.1		1.546	0.837–2.854	0.164
Operation type								
Segmental resection	129	90.7	75.7	71	< 0.001	1		
Extended resection	52	98.1	96.2	92.3		0.367	0.172–0.787	0.01
Histological type								
Adenocarcinoma	146	93.2	80.6	75.1	0.812			
Signet ring cell/mucinous	31	93.5	87.1	87.1				
Other	4	75	75	75				
Histological grade								
Grade I	25	100	96	2	0.164			
Grade II	116	92.2	80.1	74				
Grade III/IV	38	89.5	75.9	75.9				
Unclassified	2	100	100	100				
T stage								
T0	3	100	100	100	0.056			
T1	10	100	100	80				
T2	14	92.9	92.9	92.9				
T3	86	97.7	82.2	79.8				
T4	68	85.3	75	69.1				
N stage								
N0	112	94.6	89.3	84.8	< 0.001	1		
N1	48	91.7	76.4	72		1.521	0.839–2.759	0.167
N2	21	85.7	52.4	47.6		2.715	1.286–5.732	0.009
M stage								
M0	167	95.2	85.5	81.9	< 0.001	1		
M1	14	64.3	35.7	19		4.231	2.013–8.889	< 0.001

**Fig. 3:**
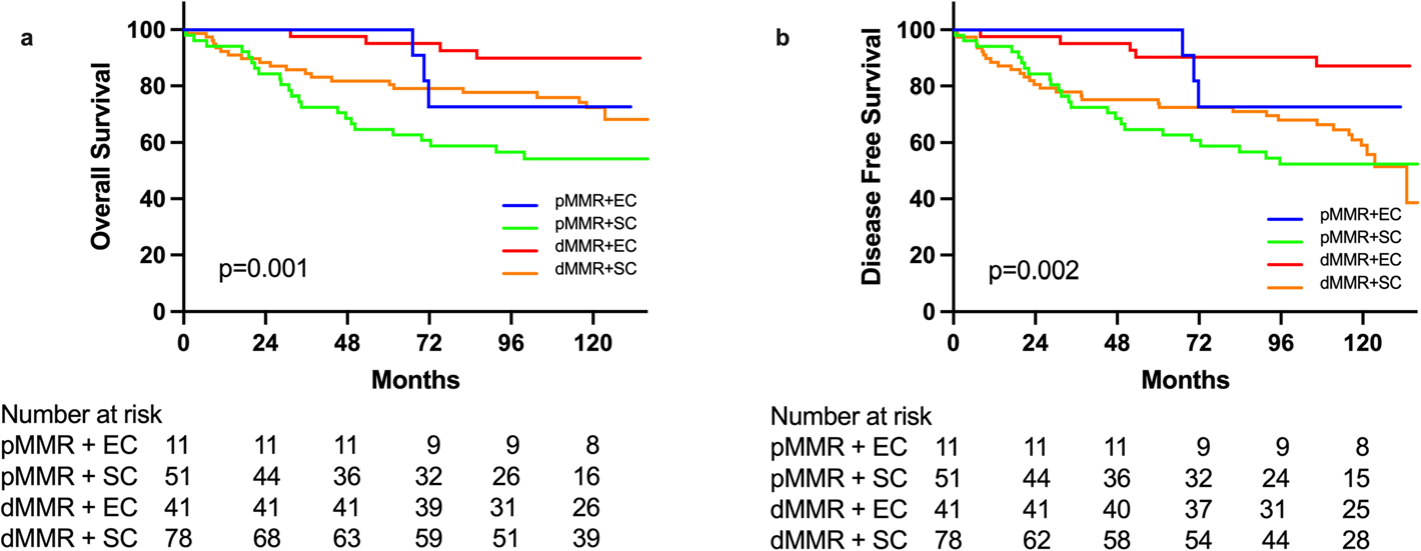
Comparison of overall survival (**a**) and disease-free survival (**b**) in HNPCC patients by combination of mismatch repair gene type and surgery type

**Fig. 4:**
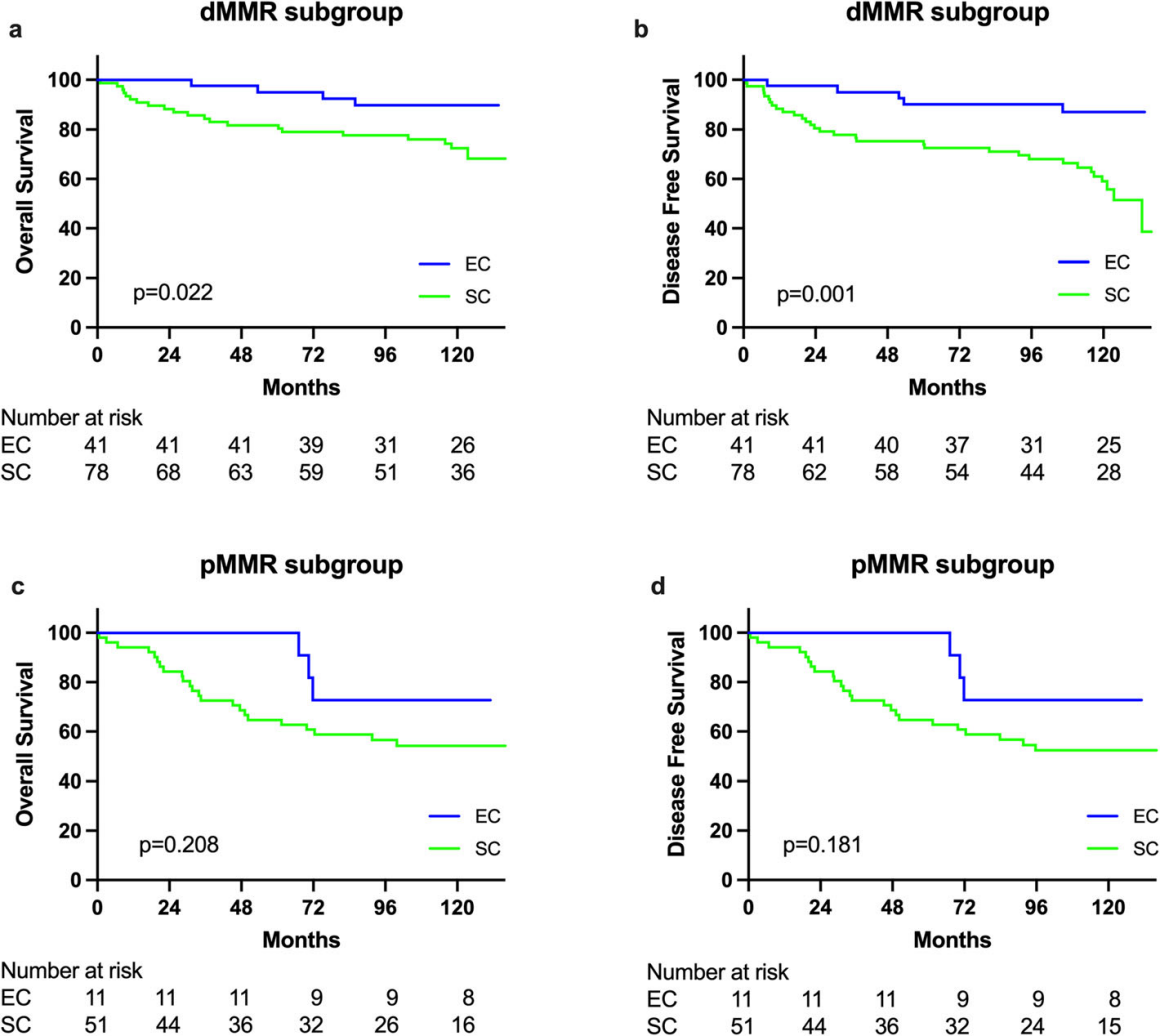
Comparison of overall survival (**a**) and disease-free survival (**b**) between patients in dMMR subgroup who had extended colectomy (EC) and those who had segmental colectomy (SC); Comparison of overall survival (**c**) and disease-free survival (**d**) between patients in pMMR subgroup who had extended colectomy (EC) and those who had segmental colectomy (SC)

We further analyzed if significant surgical outcomes exist by combing these two factors interaction (extent of resection and MMR status). Showing as Fig. 3A and B, there was significantly difference in terms of OS (*p* = 0.001) and DFS (*p* = 0.002) existing among different subgroups after combing extent of resection and MMR status (Table 6). dMMR plus extended resection showed best survival benefit in both OS and DFS while pMMR with segmental resection had worst survival (Table 6). We then analyzed dMMR and pMMR subgroups respectively, as shown in Fig. 4A and B. Shown in Table 7, in dMMR subgroup, SC, compared with EC, demonstrated significantly worse DFS by multivariate analyses (HR = 3.526, 95% CI: 1.346–9.236, *p* = 0.010) but not significantly difference for OS (HR = 2.387, 95% CI: 0.788–7.229, *p* = 0.124), however, no significantly differences of OS and DFS in pMMR subgroup between SC and EC were found.

**Table 6:** Univariate and multivariate analyses of clinicopathological features with OS and DFS among 181 HNPCC patients who underwent curative resection of CRC

Characteristics	n	OS (%)			p Value	Multiple Cox*		
		1 year	3 years	5 years		HR	95% CI	p Value
MMR with operation type								
pMMR+EC	11	100	100	100	0.001	1		
pMMR+SC	51	94.1	72.5	64.7		1.906	0.548–6.629	0.31
dMMR+EC	41	100	97.6	95.1		0.447	0.095–2.103	0.308
dMMR+SC	78	92.3	84.4	81.8		0.924	0.256–3.326	0.903
Characteristics	n	DFS (%)			p Value	Multiple Cox**		
		1 year	3 years	5 years		HR	95% CI	p Value
MMR with operation type								
pMMR+EC	11	100	100	100	0.002	1		
pMMR+SC	51	94.1	72.5	64.7		1.814	0.530–6.207	0.343
dMMR+EC	41	97.6	95.1	90.2		0.467	0.108–2.026	0.309
dMMR+SC	78	88.4	77.9	75.3		1.476	0.434–5.017	0.532

*Confounding with age, tumor location, N stage, and M stage

**Confounding with tumor location, N stage, and M stage

**Table 7:** Univariate and multivariate analyses of clinicopathological features with OS and DFS among 181 HNPCC patients who underwent curative resection for CRC, according to MMR status

	Characteristics	Univariate			Multivariate		
		HR	95% CI	p Value	HR	95% CI	p Value
OS	dMMR+EC	1			1		
	dMMR+SC	3.252	1.115–9.485	0.031	2.387	0.788–7.229	0.124
DFS	dMMR+EC	1			1		
	dMMR+SC	4.252	1.653–10.935	0.003	3.526	1.346–9.236	0.010
OS	pMMR+EC	1			1		
	pMMR+SC	2.128	0.638–7.098	0.219	1.919	0.532–6.918	0.319
DFS	pMMR+EC	1			1		
	pMMR+SC	2.219	0.667–7.377	0.193	1.991	0.555–7.140	0.291

In our cohort, there were no metachronous CRC for patients underwent EC during follow up period. However, 11 patients developing metachronous CRC in the SC subgroup (median: 34.1 months, ranging from 4.7 to 99.3 months), the positive and negative prediction rate of metachronous CRC in dMMR subgroup was 12.8 and 87.2% while 1.9 and 98.1% in the pMMR subgroup.

## Discussion

In this retrospective study, we demonstrated that among clinically defined HNPCC patients, by multivariate analyses, extended colectomy significantly improved disease free survival but no overall survival improvement compared with segmental colectomy for dMMR patients. In contrast, there is no significant difference for both DFS and OS among pMMR patients.

Previous studies recommended that EC rather than SC for the index tumor management, which become the choice of surgical treatment for LS patients because of its high risk of metachronous CRC [1, 5–11]. However, merit of EC compared with SC among clinical defined HNPCC patients became complex because HNPCC was a more heterogeneous group [20]. At least, two subgroups (dMMR and pMMR) exist in the HNPCC patients [16–18, 20]. Significantly lower risk of patients developing metachronous colorectal cancer in pMMR subgroup had been reported compared with dMMR subgroup [4, 16–18, 20]. In this study, we demonstrated EC significantly affect disease free survival in the dMMR subgroup (HR = 0.284, 95% CI: 0.108–.0743, *p* = 0.010, Fig. 4B). However, EC did not impact on DFS comparing with SC in the pMMR subgroup (Fig. 4D). Data from the present study reflected that the risk of metachronous colorectal cancer of pMMR subgroup was significantly lower than dMMR subgroup after SC (in this study, 12.8% dMMR patients in contrast to 1.9% pMMR patient underwent SC developed metachronous CRC). These findings suggested that MMR status may be considered as an important surrogate factor in HNPCC patients for making decision of operation types.

Regarding index tumor located at rectum, should we do proctocolectomy with ileoanal reservoir or low anterior resection become another question. Most surgeons would not take it lightly but they still worried about second CRC after longer follow up years. In this study, we found 40 patients with rectal cancer diagnosed, 23 (57.5%) are pMMR while 17 (42.5%) are dMMR cases. Among these patients, 3 patients developed metachronous CRC and they were all dMMR subtype of index cancer. We may well address that proctocolectomy can be considered only if rectal cancer with dMMR status was confirmed.

Although in the era of genomics, sophisticated development of genetic test makes it feasible in clinical practice, some patients still refuse to undergo genetic test or cannot afford these tests in regions without health insurance reimbursement such as in Taiwan. In this case, family history (AC-II) combined with immunohistochemistry test of MMR proteins provide a very good surrogate in practice in the real world. Preoperative testing with MMR status is important not only because it can affect surgical decision making but also its relatively cost effect benefit in clinical practice. MMR status could provide information similar to genetic data. According to a report by Quezada-Diaz et al. [21], in Lynch syndrome patients, MSH6 or PMS2 pathogenic variant could be recommended with SC and long-term colonoscopy surveillance rather than EC because no metachronous CRC detected during a 10-year follow up. However, we did not observe similar findings because in our study, 6 of 34 (17.6%) patients with MSH2/MSH6 loss and 5 of 81 (6.2%) MLH1/PMS2 loss patients developed metachronous CRC. The difference may be resulted from different population and follow up durations.

As showing in our study and others, pMMR status is more likely in cases such as older age (54.6 years old in this study) [4, 16, 18, 20], left colon predominant (30.8% in this study) [4, 16] and higher rate of rectum involved (35.4% in this study) [4, 16–18]. In these cases, SC might be considered if MMR status or genetic testing data not available. However, it is highly recommended that EC to be considered as an option in HNPCC patients with right colon tumor because of its higher rate of dMMR status or Lynch syndrome.

Age and comorbidities are two other factors should be concerned when we make EC as choice of operation, because EC had higher rate of postoperative complication and poor life of quality of these patients. In these cases, a proportion of dMMR patients may resulted from hyper-methylation of MMR genes rather than gene mutations [22]. In other words, aging patients may not be the case of lynch syndrome, moreover, there is fewer life expectancy in elderly to develop metachronous CRC. So, SC followed by close observation seems enough. However, as presented in our study, we combined family history of AC-II determined HNPCC patients and MMR status and found the average age of onset is below 50 years-old. Comorbidities are thus rare in these age group. The rate of metachronous cancer may vary from the aggressiveness of colonoscopy surveillance and the length of follow up period [23]. Moreover, AC-II positive patients with pMMR status were reported as another subgroup of familiar hereditary cancer such as FCCTX and showing relative lower rate of metachronous CRC compared with lynch syndrome [4, 16–18, 20]. We previously reported the cumulative rate of metachronous cancer in another study is 12 and 2.7% respectively for dMMR and pMMR HNPCC patients at 10 years, showing a relatively lower rate compared with previous studies showing 20% at 10 years follow up [21]. The difference of rate may be related to our aggressive postoperative surveillance strategy and metachronous cancer rate at 10 years may up to 40% in different population such as Lynch syndrome patient group [24].

This was a retrospective study, we included a clinical defined HNPCC group and the surveillance interval varied from one to three years depending on various physicians taking care policies. However, in dMMR/HNPCC patients, SC followed with surveillance strategy showed worse DFS in this study. Therefor how strict colonoscopy surveillance we should offer remained unclear for such patients because Lynch syndrome cancer was considered with a more rapid growth rate than sporadic type CRC.

In clinical setting, if young patients with small kindreds or unreliable family histories, it was difficult to apply our proposed strategy because our study based on combination of AC-II criteria and MMR status. Regarding universal MMR screening it may include some proportion of patients who resulted from hypermethlation of MMR genes not fit the case we discussed here. However, for all clinically defined HNPCC patient post-operation MMR status should be routinely checked. Because post-operative colonoscopy and extra-colonic surveillance were thus individualized based on MMR data [25]. Post-operation stringent colonoscopy surveillance of dMMR patients underwent segmental resection was recommended because metachronous CRC risk becomes an important issue while colonoscopy follow up for pMMR patients underwent segmental resection may similar to sporadic CRC because their low incidence of metachronous CRC [26]. The incidence of metachronous CRC is determined by the type of CRC and the length of follow up. In this study, only 11 patients developed metachronous CRC because of the short follow up period and inclusion of limited cases. As we reported previously **[**27**]** the frequencies of metachronous CRC in patients with sporadic CRC and HNPCC are 2.36 and 7.55 per 1000 person-year, respectively **[**27**].**

Although HNPCC patients defined by AC-II criteria clinically, we presented a different phenotypes between dMMR and pMMR subgroups in this study and it is consisted with previous literatures **[**4**,** 16**–**17**,** 20**]**. That right colon predominance (61.2% *V.S.* 33.8%) in dMMR patients while left colon (30.8% *V.S.* 24.8%) and rectal tumor predominance (35.4% *V.S.* 14.0%) in pMMR patients. The left colon and rectum predominant in pMMR patients contributed to significantly less frequency of extended colectomy (procto-colectomy or total colectomy) performed for the index tumor located at rectum or left than index tumor at right colon.

This study is limited by its retrospective nature. Although it comes from a cohort of patients with standardized data collection, providing the opportunity to compare dMMR and pMMR subgroups in HNPCC patients in terms of clinicopathologic factors and surgical outcomes, it did not answer if extra-colonic cancer can be prevented by prophylactic surgery such prophylactic hysterectomy because we have only limited cases with dMMR status developed endometrial cancers. It should also be noted that it is possible, given the multiple statistical comparisons performed in this analysis, that the noted associations could be chance findings.

## Conclusions

Among HNPCC patients, distinct clinic-pathological features existed between dMMR and pMMR subgroups. Significantly better overall survival, younger age of onset and higher rate of metachronous CRC exist in dMMR subgroup comparing with pMMR subgroup.

Although there are no survival difference in EC and SC groups in dMMR patients, EC significantly improved DFS and was thus recommended for dMMR subgroup but not for pMMR subgroup in HNPCC patients. Preoperative tumor MMR status is highly recommended for help making decision of operation types (EC or SC).

## Data Availability

Not applicable.

## Abbreviations

HNPCC: Hereditary nonpolyposis colorectal cancer
EC: Extended colectomy
SC: Segmental colectomy
CRC: Colorectal cancer
IHC: Immunohistochemical
MMR: Mismatch repair
pMMR: proficient mismatch repair
dMMR: deficient mismatch repair
LS: Lynch syndrome
